# Data-Driven Modeling of Breast Cancer Tumors Using Boolean Networks

**DOI:** 10.3389/fdata.2021.656395

**Published:** 2021-10-20

**Authors:** Domenico Sgariglia, Alessandra Jordano Conforte, Carlos Eduardo Pedreira, Luis Alfredo Vidal de Carvalho, Flavia Raquel Gonçalves Carneiro, Nicolas Carels, Fabricio Alves Barbosa da Silva

**Affiliations:** ^1^ Engenharia de Sistemas e Computação, COPPE-UFRJ, Rio de Janeiro, Brazil; ^2^ Center of Technological Development in Health (CDTS), FIOCRUZ, Riode Janeiro, Brazil; ^3^ Apoptosis Research Centre, Department of Biochemistry, School of Natural Sciences, National University of Ireland, Galway, Ireland; ^4^ Facultade de Medicina, UFRJ, Rio de Janeiro, Brazil; ^5^ Laboratório Interdisciplinar de Pesquisas Médicas– Instituto Oswaldo Cruz, FIOCRUZ, Rio de Janeiro, Brazil; ^6^ Platform of Biological System Modeling, Center of Technological Development in Health (CDTS), FIOCRUZ, Riode Janeiro, Brazil; ^7^ Scientific Computing Program (PROCC), FIOCRUZ, Rio de Janeiro, Brazil

**Keywords:** Boolean networks, cancer theranostics, systems biology of cancer, breast cancer modeling, gene regulatory network analysis

## Abstract

Cancer is a genomic disease involving various intertwined pathways with complex cross-communication links. Conceptually, this complex interconnected system forms a network, which allows one to model the dynamic behavior of the elements that characterize it to describe the entire system’s development in its various evolutionary stages of carcinogenesis. Knowing the activation or inhibition status of the genes that make up the network during its temporal evolution is necessary for the rational intervention on the critical factors for controlling the system’s dynamic evolution. In this report, we proposed a methodology for building data-driven boolean networks that model breast cancer tumors. We defined the network components and topology based on gene expression data from RNA-seq of breast cancer cell lines. We used a Boolean logic formalism to describe the network dynamics. The combination of single-cell RNA-seq and interactome data enabled us to study the dynamics of malignant subnetworks of up-regulated genes. First, we used the same Boolean function construction scheme for each network node, based on canalyzing functions. Using single-cell breast cancer datasets from The Cancer Genome Atlas, we applied a binarization algorithm. The binarized version of scRNA-seq data allowed identifying attractors specific to patients and critical genes related to each breast cancer subtype. The model proposed in this report may serve as a basis for a methodology to detect critical genes involved in malignant attractor stability, whose inhibition could have potential applications in cancer theranostics.

## Introduction

Cancer is a multifactorial disease resulting in uncontrolled cell growth and the spread of cancer cells from the original site to other body areas. The modification of cellular homeostasis through various processes identifies and characterizes the Hallmarks of cancer (Hanahan and Weinberg, 2011), typical to all types of tumors. Cell survival, proliferation, and metastatic dissemination are driven by different cellular pathways, with many genes involved. These highly complex interconnections modify the linearity of the pathways allowing the conceptualization of a reticular structure made up of genes, proteins and other molecules, characterizing cancer as a network disease. This structure defines a robust state of endogenous networks (Yuan et al., 2017; Su et al., 2017), which dynamically describes the cellular network as composed of oncogenic factors, tumor suppressors, and other acting agents, which modulate the main molecular functions.

Breast cancer, which is the type of cancer addressed in this report, is the leading cause of death due to cancer of the world’s female population. It accounts for 23% of all cancer deaths of postmenopausal women (Akram et al., 2017). Current therapies used to combat this disease frequently produce harmful side effects. In patients undergoing chemotherapy, 38 symptoms were identified, classified into 5 clusters characterizing the symptomatology (Chan et al., 2017). Therefore new therapeutic strategies aiming to decrease the undesirable effects produced by current treatment approaches, together with improved therapeutic efficacy, are needed. Personalized medicine seems to increasingly gain importance in patient care. The purpose of this therapeutic approach is to adapt the treatment to the unique characteristics of the individual patient’s disease (Sabatier et al., 2014), which are based not only on the site of the tumor but also on genetic characteristics such as mutations and gene expression profiles. There are different methodologies to model gene regulatory networks. The ordinary differential equations (ODE) and stochastic differential equations (SDE) are quantitative approaches that allow an instrumental and detailed description of the system’s dynamic functioning when the exact mechanisms and kinetic parameters are well known. Given the noise level of cellular processes, the precise determination of ODE and SDE parameters is challenging (Nasti, 2020). A qualitative approach would help avoid ODE and SDE limitations while providing useful information on the system under study. Boolean network Modeling is an example of this methodology (Somogyi and Sniegoski, 1996). It is composed of Boolean variables representing the nodes (which corresponds to vertices in a graph) making up the network, whose values are periodically updated synchronously (i.e., all nodes are updated simultaneously) or asynchronously. These updated values represent the activation/inhibition status of the genes that make up the studied system (Barbuti et al., 2020). The dynamic simulation of the network, guided by the Boolean functions that regulate the relations between the various vertices, reaches a set of final stable states, which can be cyclic or not. These repetitive states compose network attractors. The formulation of the concept of “Epigenetic Landscape” by Waddington (Waddington, 1957) offers the opportunity for modeling cellular functioning through attractor theory (Huang et al., 2009). The Boolean paradigm allows the processing and analysis of vast gene regulatory networks, resulting in an improved capacity to model the complexity of cancer since no parameters are required.

This report analyzed a gene regulatory network specifically adapted to breast cancer through a qualitative dynamic analysis using Boolean network modeling. From the choice of the network vertices (genes), the network topology, and the definition of the functional relationships at each vertex, one may found the attractors within the system through the assignment of binary gene expression values. We adopted a step-by-step network pruning approach to identify the genes being key determinants of specific basins of attraction with therapeutic relevance. Generally, when looking for attractors in a Boolean network, one considers every possible vertex configuration (Barbuti et al., 2020). On the other hand, in our approach, we identify biologically relevant attractors through trajectories. The initial point of these trajectories is the binarization of the cellular data of specific gene expression of a given tumor belonging to a given individual, enabling different and specific outcomes for different patients.

The network’s basins of attraction that emerged from the single-cell RNA-seq (scRNA-seq) data (Saliba et al., 2014) represent this research’s culmination. The essential genes that contribute to the stability of a basin of attraction can be considered potential therapeutic targets since they may modify the epigenetic landscape in which they are involved. The results described in this work show a difference between the various basins of attraction related to cancer and control cells, therefore confirming the relevance of the data-driven customization procedure based on patients’ transcriptional data. This work also describes methods for identifying potential therapeutic targets specific to each patient using boolean network modeling.

## Materials and Methods

### Overall Description of the Method

The main steps of the method adopted in this report are as follows:

1) Selection of breast cancer-related genes and subsequent gene regulatory network construction based on this gene set.
2) Adoption of the Boolean formalism for the dynamic modeling of the system and Boolean function assignment (i.e., nested canalyzing functions) to all network nodes.
3) Selection of single-cell RNA-seq (scRNA-seq) data related to breast cancer, assigning expression values to the gene regulatory network’s corresponding nodes
4) Binarization, for the set of cells in the dataset of step 3, of the expression values assigned to each gene.
5) Search for attractors in each cell provided in the dataset. We use the binarized values assigned to the network genes for each cell (step 4) as the initial value for a trajectory simulation. The set of states that compose the final cycle of the trajectory corresponds to the cell’s attractor.

This procedure allowed us to highlight attractors and related genes constantly expressed in the dataset of different patients.

This section consists of 6 subsections. In subsection *Choice of the Elements of the Gene Regulatory Network*, we describe the procedure by which we selected the constituent elements of the gene regulatory network used in this report. The description of Boolean formalism used to model the network dynamics is in subsection *Construction of the Boolean Network Model*. In subsection *Single-Cell RNA-Seq Data* we describe the scRNA-seq data used to quantify the network genes. In subsection *Binarization of scRNA-Seq Data*, we illustrate the method by which the scRNA-seq values assigned to the constituent elements of the gene network have been binarized, and describe the tool used in this report to obtain this result. The last subsection (*Search for Attractors*) describes the essential characteristics of the network’s attractors and the procedure, through an appropriate software tool, of its identification by simulating trajectory dynamics.

**FIGURE 1:**
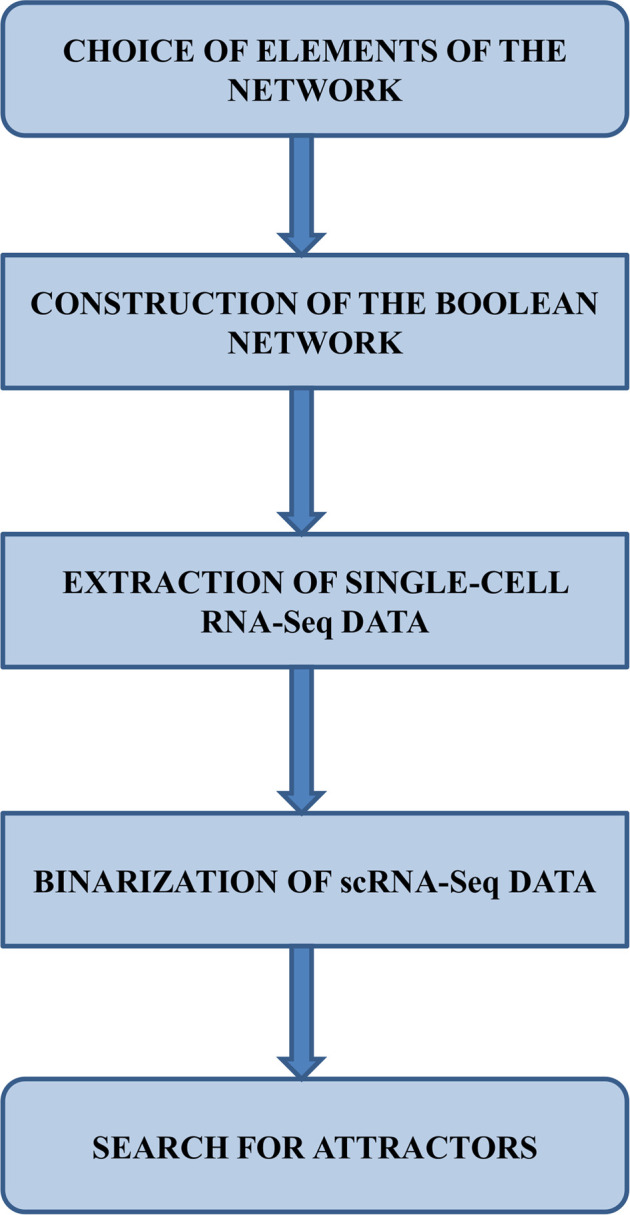
Workflow illustrating the various stages of the method used in this work.

The following subsections detail the steps shown in Figure 1.

### Choice of the Elements of the Gene Regulatory Network

Hallmarks of cancer represent groups of acquired biological features that are critical for its development (Hanahan and Weinberg, 2011). We considered two of these hallmarks, UNLIMITED REPLICATIVE POTENTIAL, and EVASION OF CELL DEATH, as starting points for constructing a representative gene regulatory network of cancer. This modeling strategy was chosen to reduce cancer cell proliferation and promote their death. We then obtained four lists of genes from the MSigDB repository (http://www.gsea-msigdb.org/gsea/msigdb/index.jsp) based on the two hallmarks previously considered, each list representing a specific cellular pathway. The gene lists related to Apoptosis and TP53 represent the “Evasion of cell death” hallmark (Wong, 2011), while Kras pathway (up and down-regulated genes) is indicative of the “Unlimited replicative potential” hallmark (Jančík et al., 2010; Aubrey et al., 2016). The choice of these pathways is justified by their particular relevance in the formation and evolution of tumors, along with the potential model scalability. We retained only the significantly differentially expressed genes (DE) between the MDA-MB-231 cell line, a metastatic triple-negative breast cancer subtype (TNBC), and the MCF10A cell line, used as control (Carels et al., 2015).

The selected genes were analyzed considering the number of interactions (edges) of their respective proteins (vertices) in the interactome. The human interactome used in this report is from the intact-micluster.txt file (IntAct database, version updated December 2017 accessed on January 11, 2018, at ftp://ftp.ebi.ac.uk/pub/databases/intact/current/psimitab/intact-micluster.txt). Proteins with edge numbers equal to or greater than 50 were selected as seeds to build the gene regulatory network. Those proteins are potential hubs, for which inhibition has been widely associated with regulatory network disruption (Carels et al., 2015).

We also added five genes to the analysis (HSP90AB1, YWHAB, VIM, CSNK2B, and TK1) (Carels et al., 2015) whose knockdown was shown to inihibit the cell growth and promote the cell death of MDA-MB-231 *in vitro* (Tilli et al., 2016).

We used the human interactome to define the connections between the proteins coded by the selected genes (network vertices). In case of a lack of a direct relationship between two vertices, we looked for possible intermediary vertices (up to three). We excluded intermediary vertices absent in the gene expression data or with low expression values in MDA-MB-231.

We enriched this network with transcriptional factors that regulate the selected vertices, i.e., differentially expressed hubs and intermediary proteins. We performed this analysis with the online tool TRRUST (Han et al., 2015).

The human interactome from IntAct defines the direction of the interactions (node A regulates node B), but not their function (activation or inhibition). For the definition of interaction functions, we used the Metacore algorithm (Ekins et al., 2007).

### Construction of the Boolean Network Model

We constructed a directed graph model based on Boolean logic from scRNA-seq data. The vertices represent the constituent elements of the dynamic cellular model, and their connections are for the functional regulations acting between them (Emmert-Streib et al., 2014). Boolean network modeling is among the simplest methods for dynamic modeling (Thomas, 1973), but at the same time with characteristics of reliability in providing insights into the dynamics of a system (Herrmann et al., 2012; Siegle et al., 2018). We have translated the gene expression status of a gene into the value of a Boolean variable ( B) , which can be True or False (1 or 0) based on RNA-seq data. Thus, for the ***n*** vertices of our network, we have:

$$X=\left\{x_{1},\;x_{2},\;x_{3},\ldots .,x_{n}\right\}\;,\;x_{i}\in B$$ (1)

This formalization finds its justification considering that one can describe many biological processes, such as concentration levels, through the Hill-Function. For most of the Hill function coefficient values, the resulting curve is a sigmoidal curve, which can be approximated by a dichotomous step-function (Schwab et al., 2020).

The representation of this network’s state in the discrete-state flow of time is a vector whose components are the network’s vertices:

$$\vec{x}=\left(x_{1}\left(t\right),\ldots \ldots ,x_{n}\left(t\right)\right)$$ (2)

and the passage from a certain point of the state space of the system to another is due to the regulatory action of the corresponding Boolean functions:

$$x_{i}\left(t+1\right)=f_{i}\left(\vec{x}\left(t\right)\right),f_{i}:B^{n}\to B$$ (3)

for ***n*** nodes of the network.

We decided to adopt a synchronous update mode, where all genes update their values simultaneously at consecutive time points:

$$T\left({\vec{x}}_{i}^{t},\;{\vec{x}}_{i}^{t+1}\right)=T_{1}\left(x_{1}^{t},x_{1}^{t+1}\right)\wedge \ldots .\wedge T_{n}\left(x_{n}^{t},x_{n}^{t+1}\right)$$ (4)

In the Eq. 4, where $T\left({\vec{x}}_{i}^{t},\;{\vec{x}}_{i}^{t+1}\right)$ represents the transition function of the state of the network, all the genes in the network simultaneously perform the transition from the state ${\vec{x}}_{i}^{t}$ to the next state $\;\;{\vec{x}}_{i}^{t+1}$ in transitions $T_{1},T_{2},\ldots ,T_{n}$ occorring in the system. Some reports state that asynchronous updating seems better to model biological systems (Schwab et al., 2020). Nevertheless, synchronous dynamic evolution is computationally more efficient for the type of network used in this report and seems to represent the network’s dynamic behavior in a very similar way (Schwab et al., 2020).

Identifying the rules of interaction among the different entities of a network is usually one of the most challenging tasks in studying gene regulatory network systems. Our choice was oriented towards the nested canalyzing functions (Hinkelmann and Jarrah, 2012), where multiple variables act simultaneously on the function, determining a mechanism of domination of one variable or a group of variables concerning the others based on their Boolean state. For example, in the expression **(A ∧ B) ⋁ (C ∧ D)**, if **A ∧ B = 1**, the first two variables dominate and determine the expression value. If **(A ∧ B) = 0**, the expression value is defined by **(C ∧ D)**. Furthermore, it has been shown that nested canalizing functions are a good representation of biological regulations (Nikolajewa et al., 2007) (Harris et al., 2002).

### Single-Cell RNA-Seq Data

The scRNA-seq data were obtained from the NCBI Gene Expression Omnibus database (accession number GSE 75688, accessed in March 2020). These data refer to the genomic expression profile of 11 patients with 549 cells analyzed. Most of those cells were malignants, while others were not. A large part of the latter were immune T-cells, immune B-cells, and myeloid immune cells. The cancer cells analyzed represented the four subtypes of breast cancer: luminal A, luminal B, HER2, and TNBC (Chung et al., 2017). We used single-cell data for the analyzed network’s corresponding genes, excluding data related to pooled samples (bulk RNA-seq) (Supplementary Table S1). The four subtypes of breast cancer were present among the samples of the 11 patients: BC01_X and BC02_X for luminal A, BC03_X for luminal B, BC04_X, BC05_X and BC06_X for HER2, BC07_X, BC08_X, BC09_X, BC10_X and BC11_X for TNBC. For BC03_X and BC07_X patients, there were metastatic lymph datasets corresponding to BC03lN and BC07LN. For the patient BC09_X, there was another single-cell RNA-seq (BC09Re_X). Note that patient BC05 is the only patient who received prior treatment (neoadjuvant chemotherapy and Herceptin).

As specified in the above description, the different types of breast cancer encountered in this report have already been identified in the dataset.

It is worth pointing out that the model is associated with a specific group of cells for each patient. Nevertheless, this approach can be conceptually made equivalent to the one defined as a multi-cell pathway, and that the relatively high number of available cells analyzed allowed a correct use of the R “Binarize” application, used in this report for the extraction of the Boolean value.

### Binarization of scRNA-Seq Data

Once the genes making up the network were found and its topology defined, and finally assigned the corresponding scRNA-seq values to each element of the network, the next operation necessary for the Boolean network modeling of the system was to binarize the gene expression values assigned to each single node, such as f:ℝ→B using

$$f\left(u\right)=\{\begin{array}{cc} 0 & u\leq t\\ 1 & u\geq t \end{array}$$ (5)

where t is the separation threshold. This result was achieved through the use of the BASC-B algorithm (Binarization Across multiple SCales) (Hopfensitz et al., 2012). The BASC algorithm considers as input values a sorted vector in ascending order $\left(u_{1},\ldots ,u_{N}\right)\in \mathbb{R}$ , and based on it, BASC defines a discrete, monotonically increasing step function ***f(x)*** with *N* steps and *N - 1* discontinuities:

$$f\left(x\right)=\sum_{i=1}^{N}u_{i}I_{A_{i}}\left(x\right)$$ (6)

with i∈{1,…,N} . Defining $d_{i}=N-1$ as discontinuities, we have $A_{i}$ as intervals defined as follow

$$A_{i}=\{\begin{array}{cc} \left(0,d_{i}\right], & if\;i=1\\ \left(d_{i-1},N\right], & if\;i=N\\ \left(d_{i-1},d_{i}\right], & otherwise \end{array}$$ (7)

and $I_{A}$ as

$$I_{A_{i}}\left(x\right)=\{\begin{array}{cc} 1, & if\;x\in A\\ 0, & otherwise \end{array}$$ (8)

Once the step function ***f(x)*** is obtained using the output vector ordered in increasing order, the algorithm calculates additional step functions that approximate this function with a smaller number of discontinuities. The algorithm then finds the strongest discontinuity in each step function and estimates the strongest discontinuities’ location and variation. This algorithm was implemented through the R Software package BiTrinA (Müssel et al., 2016).

### Search for Attractors

After defining the binarized RNA-seq values on each node of the network and establishing the rules that determine its dynamics, we sought the network’s stable equilibrium state, i.e., the attractors, which can be either singleton (composed of a single state) or cyclic (composed of multiple states) (Huang et al., 2009). The hypothesis under which one may consider the malignant state as a particular type of attractor (Huang et al., 2009; Creixell et al., 2012; Yu and Wang, 2016; Poret and Guziolowski, 2018) has oriented our investigations towards the localization and characterization of attractors in Boolean networks. Furthermore, basins of attraction include all the system states that evolve into a given attractor. They conceptually represent the epigenetic barriers that delimit the basin of attraction (Conforte et al., 2020). We obtained the corresponding attractors matching the gene network for each scRNA-seq dataset of the eleven patients with breast cancer (Chung et al., 2017). Attractor analysis allowed us to highlight the key genes in each basin of attraction and how their inhibition could determine a change in cell fate by using the python Open Source software application “BooleanNet” (Albert et al., 2008).

We performed the following procedure to search for attractors from the available data:

• We used BooleanNet (Albert et al., 2008) to assess the logic functions assigned to each gene of our regulatory network and search for Boolean attractors.
• The Boolean values of the 103 genes making up the network were obtained by the binarization of scRNA-seq relative to each patient sample. This setting was the initial state of a trajectory that eventually evolved to a cyclic attractor.
• Considering that all the attractors obtained were cyclical for each cell analyzed, we assessed the behavior of every single gene in the network by noting whether they varied in their boolean value during the attractor cycle or if they kept a fixed Boolean value for the entire attractor cycle. In the first case, we indicated genes in each particular cell with an “X,” in the second case with its Boolean value True or False.
• By grouping all cells according to their batch samples (BCXX_X) and their carcinogenic features for each patient, we selected only the genes that did not show variations in boolean values in any of the attractors for all cells, i.e., we kept their Boolean value True or False in most states of the attractor cycle, for at least 95% of the number of cells making up the group under analysis.

**FIGURE 2:**
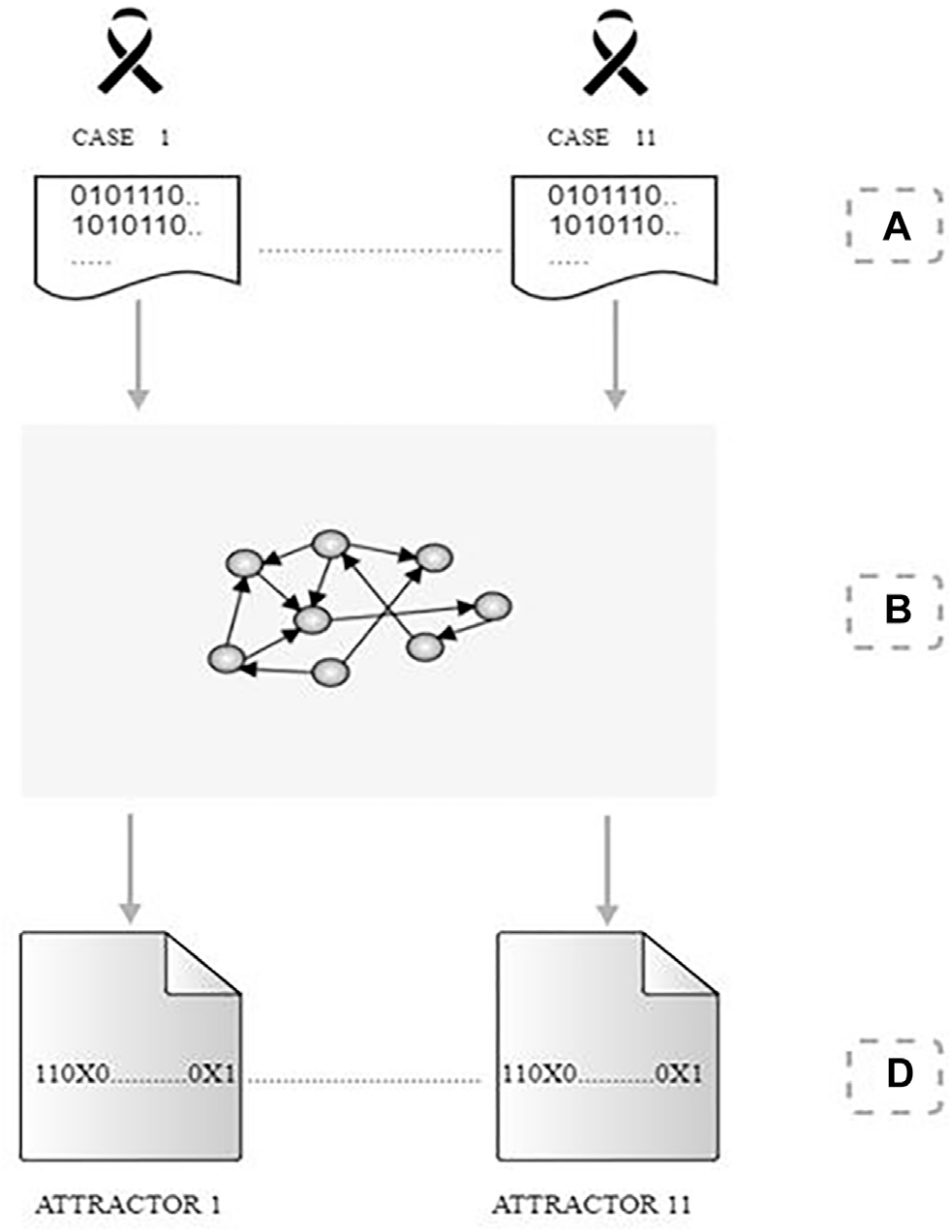
Procedure for identifying attractors. **(A)** we obtain a set of Boolean values for the cell samples of 11 patients considering a regulatory network of 105 genes. **(B)** Each patient’s Boolean data was processed individually in the gene network to search for cell attractors. **(C)** For each detected attractor, the genes that did not change their Boolean value for the set of states that compose the cyclic attractor received the value “True” or “False” (1 or 0). The marker “X,” on the other hand, highlights the genes that did not keep a single Boolean value in the set of states of the cyclic attractor.

Figure 2 summarizes the adopted procedure.

## Results

### Breast Cancer Gene Regulatory Network

The process of choosing the elements (genes) constituting the gene regulatory network adopted in this report produced the following results.

First, we obtained 761 genes derived from the Broad Institute repository, divided into four lists related to the two cancer hallmarks used to build the network. The 761 genes were classified as follows (Supplementary Table S2):

• 161 related to the APOPTOSIS pathway
• 200 related to the TP53 pathway
• 200 related to the KRAS UP pathway
• 200 related to the KRAS DOWN pathway.

In order to retain only differentially expressed genes, we compared the lists obtained with the RNA-seq data of the MDA-MB-231 and MCF10A cell lines (Tilli et al., 2016), obtaining the following results:

• Because they were neither present in the gene expression data of MDA-MB-231 nor in the MCF10A one 1) 129 genes were excluded from the APOPTOSIS pathway list, leaving 32 genes;
• 2) 191 genes were excluded from the KRAS_UP pathway list, leaving 9 genes;
• 3) 192 genes were excluded from the KRAS_DN pathway list, leaving 8 genes;
• 4) 164 genes were excluded from the TP53 pathway list, leaving 36 genes.

Among the genes retained, we selected only those that were differentially expressed, which resulted in a total of 51 genes (Supplementary Table S3):

• 18 genes of the APOPTOSIS group, 9 Up and 9 Down;
• 7 genes of the KRAS_UP group, 3 Up and 4 Down;
• 4 genes of the KRAS_DN group, 0 Up and 4 Down;
• 22 genes of the TP53 group, 10 Up and 12 Down.

**FIGURE 3:**
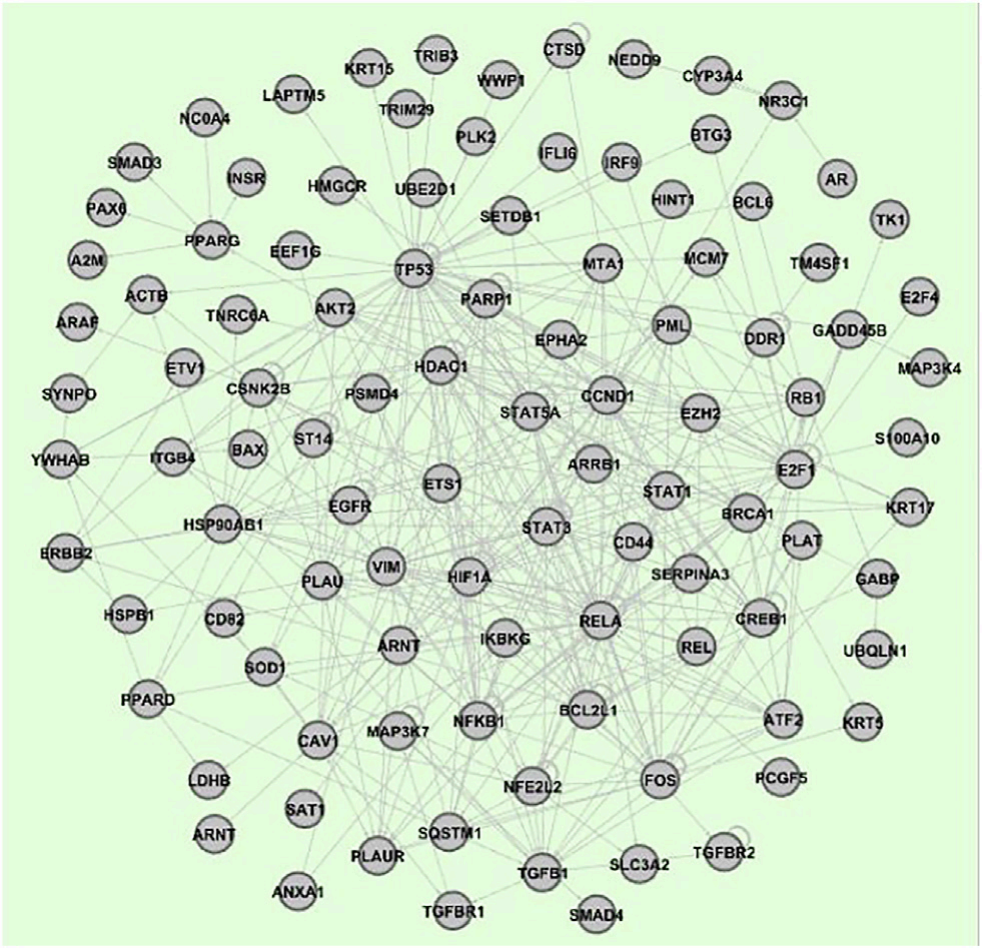
Breast cancer gene regulatory network developed in this report. This network is composed by 103 nodes (genes).

Based on the number of interactions in the interactome, 15 genes of the 51 were selected, from which 5 (*HSP90AB1, YWHAB, VIM, CSNK2B, TK1*), considered more relevant for the present research, have been added to the network (Carels et al., 2015). As outlined above, 1) the vertice vertex connections obtained by comparison with IntAct human interactome, 2) the inclusion of intermediate vertices, 3) the enrichment of the network with transcriptional factors that regulate the selected vertices with the online tool TRRUST (Han et al., 2015), and 4) the activation or inhibition of vertex inputs obtained with the Metacore algorithm (Karin, 2006) (Supplementary Table S4), allowed us to obtain a gene regulatory network consisting of 103 vertices (see Figure 3), and whose dynamics were regulated by nested canalyzing functions (Hinkelmann and Jarrah, 2012) (Supplementary Table S5).

### Binarization of scRNA-Seq Values

**FIGURE 4:**
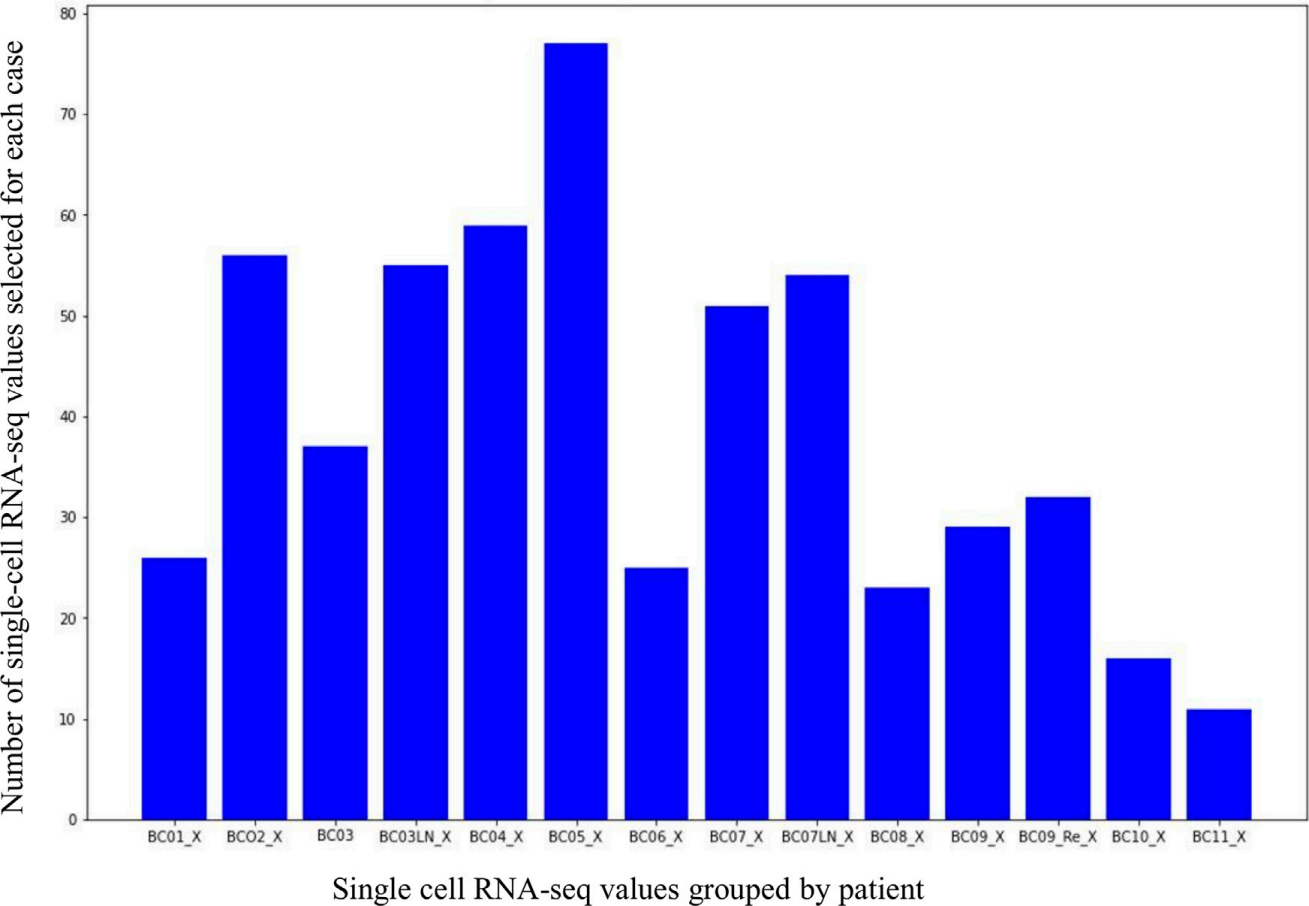
Distribution of the scRNA-seq data for each patient.

The 14 groups of scRNA-seq binarization values from the 11 patients belong to 4 types of breast cancer. They were divided and organized according to the following criterion: 26 single-cell datasets for the patient BC01_X, 56 for BC02_X, 37 and 55 for BC03_X and BC03LN, 59 for BC04_X, 77 for BC05_X, 25 for BC06_X, 51 and 53 for BC07_X and BC07LN, 23 for BC08_X, 29 and 31 for BC09_X and BC09Re, 16 for BC10_X, 11 for BC11_X (see Figure 4). It is worth noting that the values relating to pooled single-cell present in each patient group were excluded from the count.

The gene expression values of every single cell of each patient were matched to the corresponding 103 genes making up the gene regulatory network and subsequently binarized using the BASC-B algorithm (Hopfensitz et al., 2012) (Supplementary Table S6).

### Attractors Search

For every single cell of the 14 groups representing the 11 patients of breast cancer, the 103 binarized values at each node of the gene regulatory network were processed by the previously described Boolean attractor search procedure (Albert et al., 2008). The attractors obtained for malignant cells, stromal cells, immune B and T-cells, and myeloid cells are thus summarized as follow: 1) BC01_X: 19 malignant attractors, 2 stromal cell attractors, 5 no result; 2) BC02_X: 49 malignant attractors, 7 no result; 3) BC03_X: 15 malignant attractors, 7 immune B-cell attractors, 5 immune T-cell attractors, 10 no results; 4) BC03LN_X: 6 malignant attractors, 35 immune B-cell attractors, 3 immune T-cell attractors, 11 no results; 5) BC04_X: 42 malignant attractors, 3 immune T-cell attractors, 2 immune Myeloid attractors, 12 no results; 6) BC05_X: 74 malignant attractors, 3 no results; 7) BC06_X: 6 malignant attractors, 2 stromal cell attractors, 6 immune B-cell attractors, 11 no results 8) BC07_X: 24 malignant attractors, 4 stromal cell attractors, 3 immune B-cell attractors, 4 immune T-cell attractors, 8 immune myeloid attractors, 8 no results; 9) BC07LN_X: 24 malignant attractors, 19 immune B-cell attractors, 10 no results; 10) BC08_X: 15 malignant attractors, 6 stromal cell attractors, 2 no results; 11) BC09_X: 2 stromal cell attractors, 1 immune B-cell attractors, 7 immune T-cell attractors, 15 immune myeloid attractors, 4 no results; 12) BC09Re_X: 2 stromal cell attractors, 1 immune B-cell attractors, 20 immune T-cell attractors, 6 immune myeloid attractors, 2 no results; 13) BC10_X: 11 malignant attractors, 2 stromal cell attractors, 2 immune myeloid attractors, 1 no results; and 14) BC11_X: 10 malignant attractors, 1 no results.

Based on these results, we decided to exclude patient data BC09_X and BC09Re due to the lack of specific tumor cell attractors (Supplementary Table S7).

**FIGURE 5:**
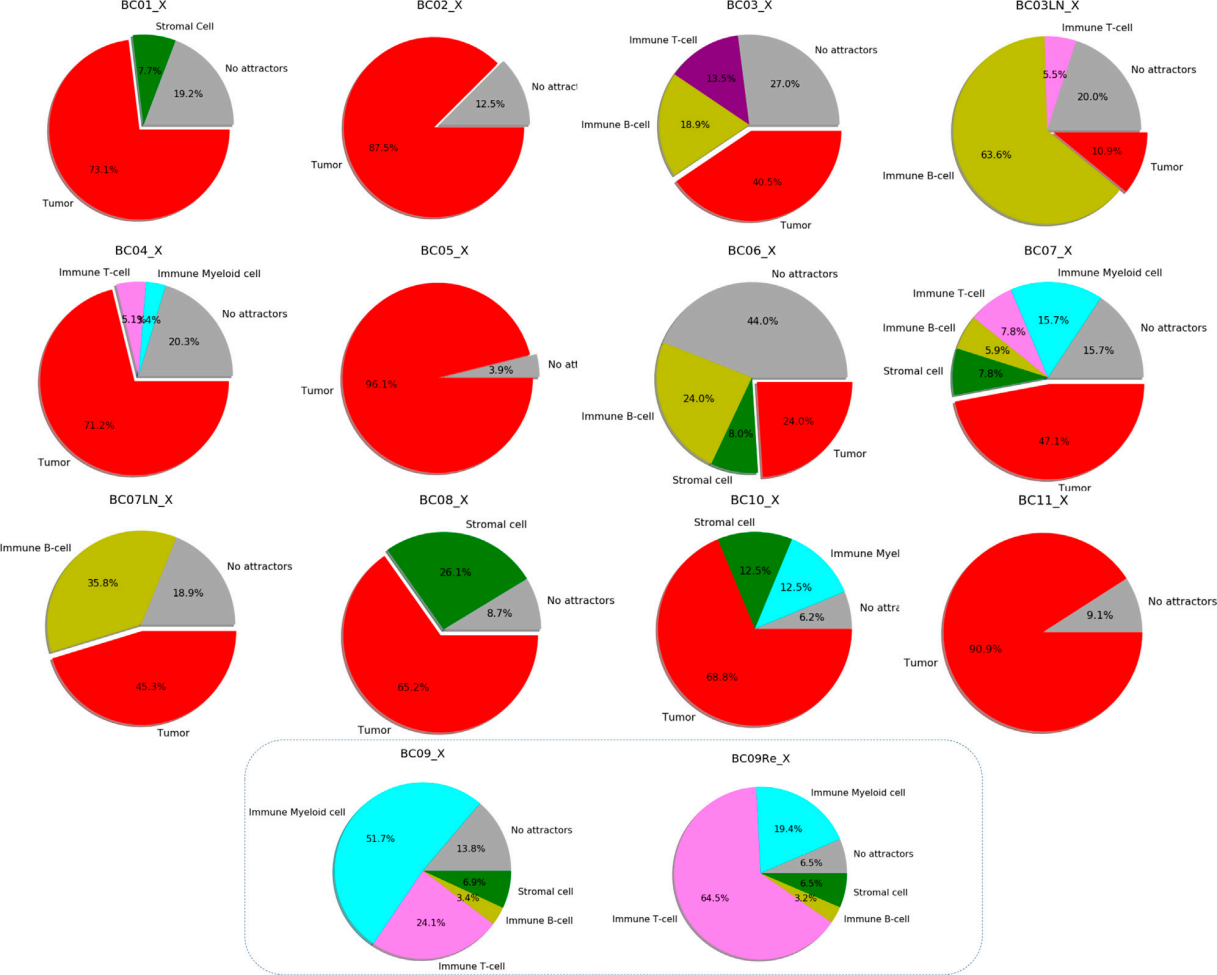
Specific categories of attractors representing a group of scRNA-seq data belonging to each patient’s breast cancer sample. Every diagram shows the percentages of attractors encountered based on the total number of cells analyzed for different cell types. The different colors refer to the type of cells analyzed: red for cancer, green for stromal cells, yellow for immune B-cells, violet for immune T-cells, cyan for myeloid immune cells. Gray indicates the percentage of cells in which it was not possible to find any attractors. The last two pie charts do not indicate tumor type attractors (absence of red color).

The pie-charts in Figure 5 shows these results expressed as a percentage of the total Single-cell RNA-seq datasets available for each patient, highlighting the success rate in the search for specific attractors on tumor cells.

We selected network genes based on tumor cells’ attractors according to the following criterion: each patient’s attractors kept their level of gene expression (or non-expression) constant for a particular gene, formalized respectively with the symbol “True” or “False”. This criterion allowed us to formulate the following considerations on the results obtained:

• BAX is expressed in the attractors of all patients except BC03_X.
• EGFR is expressed in tumor cells’ attractors for all patients except BC03_X and BC05_X.
• ERBB2 is expressed in the attractors of all patients, except BC03_X.
• ETV1 is expressed in the attractors of tumor cells for every patient, except BC03_X.
• IKBKG is expressed in the tumor attractors of all patients.
• MAP3K7 is expressed in the attractors of all patients except BC01_X.
• ST14 is expressed in the attractors of all patients except BC01_X.
• PLAT is expressed in patient attractors BC02_X, BC03_X, BC07_X, BC07LN_X, BC08_X, BC10_X, BC11_X.
• DDR1 is expressed in the tumor attractor of patient BC04_X.

**FIGURE 6:**
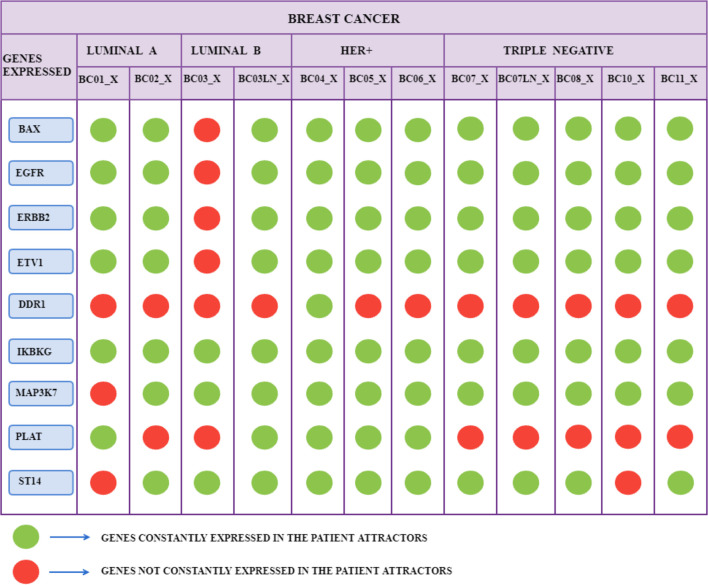
The 12 scRNA-seq groups of breast cancer samples of 10 patients, translated into attractors, are divided into four subtypes of tumor: Luminal A and B, HER+, and TNBC. The green color indicates that the corresponding gene has a constant Boolean value (True or False) for all the patient’s attractors. The red color indicates that the state of the gene does not remain constant for all the attractors of a specific patient.

These results are summarized in Figure 6.

Interestingly, PLAT is never expressed in patients with breast cancer classified as TNBC. Unlike for patients of Luminal A and Luminal B in which the inactivity of PLAT affects 50% of patients, this characteristic covers all TNBC group cases (Figure 5).

Further considerations concern the comparison between the attractors of malignant cells with other types of cells from the same patient. For example, in the BC07LN_ patient sample, it is interesting to compare EEF1G in malignant and immune B-cells. In the first case, the gene is expressed in only 4.2% of the attractors detected (1/24), while in the second case, it is expressed in 36.9% of the attractors detected (7/19). Considering DDR1, the attractor rate of expression was 60% (9/15) in the patient sample BC08_X. For stromal cells, the attractor level of expression for the same gene is 100% (6/6).

## Discussion

The widely spread use of Boolean networks to model gene regulatory network dynamics is well-established in the scientific community. Identifying attractors with this type of model enables the elucidation of long-term cell functioning, which corresponds to a particular phenotype in molecular biology. An attractor is a stable state of the cell. Starting from an initial point of the state space, the cell dynamics simulated by the model induce a sequence of states driven by the regulatory interaction among the network nodes until reaching an equilibrium. This stable set of states manifests itself with the repetition of the configuration of the network in its Boolean values in a fixed or cyclical way. Thus, the initial point from where the trajectory started is part of the basin of attraction of a given attractor in which all the points (or state spaces) contained in it converge.

This work’s central hypothesis is the interpretation of cancer phenotypes as basins of attraction in the epigenetic landscape (Huang et al., 2009). Another central assumption is that the perturbation of a subset of genes can produce the transition from one basin of attraction to another, which corresponds to another phenotype (Crespo et al., 2013). Therefore, we modeled the dynamics of breast cancer through the identification and description of the attractors associated with a specific gene regulatory network in such a way as to be able to find out the essential genes that determine the formation of the basin of attraction. These essential genes are potential therapeutic targets.

In large gene regulatory networks (with more than 100 vertices, such as the one presented in this work), it is possible to adopt different approaches to define attractors to overcome the exponential growth of the state space size according to the increase in the number of network size. For example, one approach was to partition the network into small subnets, finding the attractors corresponding to these partitions and then combining them to build a stable state relative to the entire network (Hong et al., 2015). Another approach is to configure network input vertices with initial Boolean values representing their gene expression level (Cho et al., 2016).

This report searched the network attractors resulting from the topological features and logical functions attributed to each vertex, given the binarized scRNA-seq data available. The use of single-cell data, allows a better description of the heterogeneous nature of cancer with a consequent better therapeutic outcome, unlike Bulk RNA-seq data which provide average expression levels of a cell population that may include tumor cells and other cell types. The attribution of a specific Boolean value to each vertex of the network, obtained through gene expression binarization, conditioned the initial conditions in the system’s state space. These initial conditions were the starting points of a trajectory, driven by the topology and the logic functions characterizing the network, whose evolution ended when reaching an attractor. This strategy is very time efficient, avoiding the nonpolynomial complexity of other strategies to find network attractors (Hong et al., 2015). The result obtained can be considered satisfactory given the percentage of attractors obtained.

From the analysis of the attractors obtained in this work, we extracted peculiar characteristics on several genes, demonstrating the need for a theranostics approach based on specific patient data. Key genes frequently expressed in attractors identified in this report were cited in the scientific literature related to breast cancer. They are BAX (Sturm et al., 2000), DDR1 (Belfiore et al., 2018), EGFR (Bhargava et al., 2005), ERBB2(Vernimmen et al., 2003), ETV1 (Ouyang et al., 2015), MAP3K7(Zhou et al., 2017), PLAT (Theillet et al., 1993), ST14 (Kauppinen et al., 2010).

BAX pro-apoptotic protein is differentially expressed in breast tumors by the BAX gene. The tumor suppressor gene TP53 regulates the expression of BAX and its mediated apoptosis. A reduced level of BAX expression is an adverse prognostic factor in breast cancer (Sturm et al., 2000).

DDR1, a non-integrin collagen tyrosine kinase receptor, plays an essential role in cellular communication with the microenvironment. It is differentially expressed in several malignant tumors, playing an essential role in tumor progression, including breast cancer (Belfiore et al., 2018).

EGFR is an epidermal growth factor receptor protein. It is part of pathways that control several key biological processes like angiogenesis, cellular proliferation, and apoptosis avoidance. Indeed, it is worth highlighting the FDA approved GEFITINIB availability, an anticancer drug that acts as an EGFR tyrosine kinase inhibitor. In a sample of 175 breast cancer cases, there was EGFR amplification in 11 of them. On these 11, 10 (91%) had an EGFR protein overexpression detected by immunohistochemistry (Bhargava et al., 2005).

ERBB2, commonly referred to as HER2, encodes a member of the epidermal growth factor (EGF) receptor, a family of tyrosine kinase receptors. About 30% of invasive breast carcinomas overexpress this gene and are correlated with poor prognosis. HER2 encodes a 185 kDa transmembrane receptor belonging to the EGFR group. The monoclonal antibody Trastuzumab effectively inhibits the growth of breast cancer tumors that overexpressed HER2 (Vernimmen et al., 2003).

The ETV1 protein (together with ETV4 and ETV5) forms the PEA3 subfamily of ETS transcription factors. The PEA3 group could be a tumorigenic factor in breast cancer. ETV1 expression is higher in TNBC tissues compared to normal tissues. Negative regulation of ETV1 can activate COP1 as a tumor suppressor in patients with TNBC (Ouyang et al., 2015).

MAP3K7 is an enzyme that is encoded by the MAP3K7 gene. This protein controls a series of cell functions like apoptosis and transcription regulation. Cell growth assessment performed by MTT assay showed an increase in MAP3K7 expression in breast cancer tissues compared with non-malignant breast tissue (Zhou et al., 2017). Given the crucial role of this protein in other types of cancer (Rodrigues et al., 2015; Cheng et al., 2019; Washino et al., 2019), it would be interesting investigate in more detail its role in breast cancer.

PLAT encodes tissue-type plasminogen activator, a serine protease that transforms the proenzyme plasminogen to plasmin, an enzyme. Reports in the literature point out the amplification of PLAT in breast cancer. Literature reports indicate that 15.6% of breast cancer tumors present PLAT amplification (Theillet et al., 1993). It is also interesting to note the impact on gene expression related to migration and invasion in breast cancer, especially PLAT, obtained from docosahexaenoic acid (DHA), which emerged in a recent study (Chénais et al., 2020).

ST14 encodes a matriptase protein. It is an epithelial-derived integral serine protease. The overexpression of this protein is associated with low tumor survival in node-negative breast cancer cases. It also seems that a coordinated overexpression of ST14 and other genes (MNP and MST1R) is associated with metastasis and poor breast tumor prognosis (Kauppinen et al., 2010).

IKBKG gene encodes the NF-KAPPA-B essential modulator (NEMO), a protein that is the regulatory subunit of the IKB kinase complex’s inhibitor. This protein’s overexpression may occur in cases of *inflammatory breast cancer* (IBC), a rare form of breast cancer characterized by a particular phenotype (Lerebours et al., 2008). As this protein is often highlighted in the literature for its role as a growth and progression factor in several types of cancer (Karin, 2006), it might be appropriate to thoroughly investigate its role in breast cancer development.

All those scientific evidence confirm the effectiveness of the approach proposed in this work to identify biomarkers and potential therapeutic targets. The present report also produced a detailed list of genes never expressed in the attractors obtained. An example is the ANXA1 never expressed in the attractors related to the breast cancer sample BC07_X. The overexpression of the protein produced by ANXA1 seems to indicate poor overall survival in TNBC (Gibbs and Vishwanatha, 2018). Another example is the SMAD4, which is never expressed in the attractors of BC02_X. The protein produced by this gene is part of the SMAD family of transcription factor proteins, which acts on the TGF-β signal transduction. SMAD4 expression was lower in breast cancer tissue than in the surrounding breast epithelium (Stuelten et al., 2006). These constantly not-expressed genes in tumor attractors can be used as biomarkers for diagnostics, predictive, and prognostic purposes (Lerebours et al., 2008), awaiting further research advances on the challenge of increasing gene-level expression using CRISPR techniques (Matharu et al., 2019).

It is worth noting that even if we based the choice of genes that compose the network on differentially expressed genes from the MDA-MB-231 cell line, which is associated with the TNBC subtype, we succeed in obtaining attractors for other cancer subtypes as well. This result indicates that the method used to include intermediary vertices from the human interactome and related transcription factors is robust enough to capture key genes possibly involved in all major breast cancer subtypes.

It is significant to highlight another point that emerged in this report: the ERBB2 gene is a therapeutic target in breast cancer for which specific drugs exist (Gomez et al., 2008). ERBB2 is constantly expressed in all patients analyzed in this report except for one, the patient BC03-X. For this reason, BC03-X might not need the type of therapeutic intervention related to ERBB2. Such consideration allows us to place our method in the context of personalized medicine. Nevertheless, further specific algorithm development for defining the more appropriate therapeutic decision for each patient is needed.

Different settings in specific parts of the procedure illustrated in this report may be further studied. For example, one example of future work is to compare the results obtained with both asynchronous and synchronous models on the network dynamics. Another example of future work is to use specific logic functions for each node of the network instead of the nested canalyzing function approach used in this analysis.

## Conclusion

In this work, we model the complex dynamics of a gene regulatory system related to breast cancer using scRNA-seq data. We computed the attractors of the analyzed cells, as well as the genes related to attractor stability. Each group of cells belongs to a different patient, and a certain degree of differentiation between the various patients was found in the genes characterizing the attractors. This characterization drives therapeutic actions differentiated from patient to patient based on the analysis that emerged. These considerations allow us to frame the system developed in this report within the paradigm of personalized medicine. This work can be expanded in many ways. One significant advancement will be to define an algorithm to define optimal therapeutic interventions based on the analysis of the model. One crucial optimization parameter for this algorithm is to minimize the number of therapeutic interventions while providing maximum efficacy. Another contribution is to evaluate if asynchronous boolean modeling can provide new insights compared to synchronous boolean modeling. Our group intends to explore those questions soon.

## Data Availability

Source code and data used in this study are available at https://github.com/Domenico321/attractors-search.

## Supplementary Material

Supplementary Table 1 Dataset containing the scRNA-seq values used in this report.

Supplementary Table 2 The four pathway lists related to the two cancer Hallmarks used to build the network and the five additional nodes included.

Supplementary Table 3 Selection of differentially expressed genes and the number of connections related to each of them.

Supplementary Table 4 Scheme summarizing the type of relationship (activation or inhibition) existing between the various components of the network.

Supplementary Table 5 Description of the direct functional relationship between the various nodes of the network.

Supplementary Table 6 Binarized scRNA-seq values.

Supplementary Table 7 Summary table of the attractors detected, divided by patients and their cells of belonging(rows) and genes constituting the analyzed network(columns). The False value indicates that this gene, in the attractor of that cell, is always unexpressed, the True value indicates constant expression in this attractor, X indicates that its expression level varies; it is not constant.
